# Infection of Female BWF1 Lupus Mice with Malaria Parasite Attenuates B Cell Autoreactivity by Modulating the CXCL12/CXCR4 Axis and Its Downstream Signals PI3K/AKT, NFκB and ERK

**DOI:** 10.1371/journal.pone.0125340

**Published:** 2015-04-24

**Authors:** Gamal Badr, Ayat Sayed, Mostafa A. Abdel-Maksoud, Amany O. Mohamed, Azza El-Amir, Fathy A. Abdel-Ghaffar, Saleh Al-Quraishy, Mohamed H. Mahmoud

**Affiliations:** 1 Laboratory of Immunology & Molecular Physiology, Zoology Department, Faculty of Science, Assiut University, Assiut, Egypt; 2 Department of Biochemistry, Faculty of Medicine, Assiut University, Assiut, Egypt; 3 Zoology Department, Faculty of Science, Cairo University, Cairo, Egypt; 4 Zoology Department, College of Science, King Saud University, Riyadh, Saudi Arabia; 5 Deanship of Scientific Research, King Saud University, Riyadh, Saudi Arabia; 6 Food Science and Nutrition Department, National Research Center, Dokki, Cairo, Egypt; INRS, CANADA

## Abstract

Systemic lupus erythematosus (SLE) is a prototypic autoimmune disease characterized by abnormal autoreactivity in B cells. Lymphocytes and their soluble mediators contribute to the disease pathogenesis. We recently demonstrated that infecting lupus mice with malaria confers protection against lupus nephritis by attenuating oxidative stress in both liver and kidney tissues. In the current study, we further investigated B cell autoreactivity in female BWF1 lupus mice after infection with either live or gamma-irradiated malaria, using ELISA, flow cytometry and Western blot analysis. The lupus mice exhibited a significant elevation in plasma levels of IL-4, IL-6, IL-7, IL-12, IL-17, IFN-α, IFN-γ, TGF-β, BAFF and APRIL and a marked elevation of IgG2a, IgG3 and ant-dsDNA autoantibodies compared with normal healthy mice. Infecting lupus mice with live but not gamma-irradiated malaria parasite partially and significantly restored the levels of the soluble mediators that contribute to the progression of lupus. Furthermore, the B cells of lupus mice exhibited an increased proliferative capacity; aberrant overexpression of the chemokine receptor CXCR4; and a marked elevation in responsiveness to their cognate ligand (CXCL12) via aberrant activation of the PI3K/AKT, NFκB and ERK signaling pathways. Interestingly, infecting lupus mice with live but not gamma-irradiated malaria parasite restored a normal proliferative capacity, surface expression of CXCR4 and B cell response to CXCL-12. Taken together, our data present interesting findings that clarify, for the first time, the molecular mechanisms of how infection of lupus mice with malaria parasite controls B cell autoreactivity and thus confers protection against lupus severity.

## Introduction

Systemic lupus erythematosus (SLE) is a chronic multisystem autoimmune disease that is characterized by abnormal B cell activation and differentiation [1], a loss of tolerance to nucleic acids and their associated proteins and the production of autoantibodies that cause tissue damage [2]. Several studies have reported that B lymphocytes play an essential role in autoantibody production, working as antigen-presenting cells (APCs) and as a source of cytokines [3]. Because B cells play a crucial role in SLE pathogenesis, successful treatment strategies for the disease should optimally target these cell types. In SLE patients, certain cytokines and chemokines are essential mediators of pathogenesis and disease progression [4–7]. Previous reports have indicated that Th1- and Th2-type cytokines are implicated in SLE disease activity [4, 5]. In animal models, our previous study showed elevated levels of TNF-α and IL-10 in BWF1 lupus mice [6]. Recently, published data have revealed that the pro-inflammatory adaptive cytokines (types Th1, Th2, and Th17) and shed TNF receptors in SLE patients are elevated prior to disease flares, while regulatory mediators are elevated during periods of stable disease [7]. IL-7 plays several fundamental roles in B cell development by aiding in the specification and commitment of cells to the B lineage, the proliferation and survival of B cell progenitors, and the maturation of pro-B cells to pre-B cells [8]. Additionally, a recent study reported that the soluble form of the IL-7 receptor is a marker of SLE disease activity, especially nephritis [9]. Two members of the tumor necrosis factor (TNF) family, BAFF (B cell activating factor of the TNF family) and APRIL (a proliferation-inducing ligand), are already known for their crucial roles in normal B cell survival, differentiation and apoptosis and have recently been shown to be expressed by B-CLL cells [10]. In this context, systemic activation of the immune system induces aberrant BAFF and APRIL expression in B cells in patients with SLE [11]. Previous reports have described the IgG subclass concentration profile in sera from patients with SLE and revealed increased concentrations of all IgG subclass constituents [12]. However, other reports found low levels of IgG2, elevated levels of IgG1 and IgG3 and normal levels of IgG4 in patients with SLE [13]. Additionally, previous studies have revealed that murine SLE is characterized by high levels of the IgG2a and IgG3 autoantibodies, which cause glomerulonephritis [14, 15]. Chemokines and their receptors are crucial for chemotaxis, lymphocyte homing to secondary lymphoid organs and, subsequently, Ag recognition [16, 17]. We previously demonstrated that human B cells exhibit a marked surface expression of CCR6, CCR7, CXCR4 and CXCR5 and migrate toward their cognate ligands CCL20, CCL21, CXCL12 and CXCL13, respectively [18]. Chemokine-mediated B cell activation and movement is a complex phenomenon primarily driven by actin polymerization and cytoskeleton reorganization [19]. In human patients and animal models of SLE, the accumulation of immune cells at inflammatory sites, impaired immune cell functions, and migratory disturbances are due to the altered expression of several chemokine receptors on immune cell surfaces [20]. In patients with SLE, an up-regulation of CXCR4 has been reported, suggesting that the CXCR4/CXCL12 axis might be a therapeutic target for SLE patients with kidney and/or central nervous system involvement [21]. Inversely, a previous investigation revealed a down-regulation of the CXCL12 receptor (CXCR4) in circulating B cells from SLE patients, leading to their altered migratory behavior and distribution of the B cell compartment [22]. The signaling cascades involving PI3K/AKT, MAPKs (ERK, JNK, p38) and the regulation of NF-κB nuclear translocation (IκBs) are critically involved in B cell differentiation and the production of autoantibodies during SLE disease progression [23]. Moreover, aberrations in these signaling pathways lead to or are associated with autoimmune diseases such as SLE [24]. Our previous study revealed that infecting lupus mice with malaria parasite confers protection against lupus nephritis via altering the redox state in the kidney and the liver [6]. Furthermore, epidemiological studies have revealed that SLE is rarely observed where infection with malaria parasites is prevalent [25, 26] and that young lupus-prone mice have a higher survival rate when infected with *Plasmodium berghei yoelii* [27]. Moreover, the injection of immunoglobulins isolated from *P*. *chabaudi*-infected BALB/c mice produced protective effects that were to the infection itself in BWF1 mice [28]. However, few studies have investigated the effect of infection with malarial parasites on B cell biology in lupus mice. Therefore, in the current study, we investigated the possible effects of infection with *P*. *chabaudi* on the plasma cytokine profile and on B cell biology in term of autoreactivity, chemotaxis, proliferation and signaling pathways in a murine model of SLE.

## Materials and Methods

### Animals

A total of 30 experimental female BWF1 29-week-old mice (purchased from Jackson Laboratories, Bar Harbor, USA) and 10 normal non-lupus mice were maintained and monitored in a specific pathogen-free environment. All animals were allowed to acclimatize in plastic cages (5 animals per cage) inside a well-ventilated room for 1 week prior to the experiment. The animals were maintained under standard laboratory conditions (temperature of 23°C, relative humidity of 60–70%, and a 12-hour light/dark cycle), fed a diet of standard commercial pellets, and given water *ad libitum*.

### Ethics Statement

This ethical committee is approved by U.S. Department of Health and Human Services (HHS) Institutional Review Board (IRB). IORG number: IORG0006563. OMB number: 0990–0279. All animal procedures were performed in accordance with the standards set forth in the Guidelines for the Care and Use of Experimental Animals by the Committee for the Purpose of Control and Supervision of Experiments on Animals. The study protocol was approved by the Animal Ethics Committee at Cairo University.

### Infection with malaria parasite

The blood stage forms of *P*. *chabaudi* parasites were stored in liquid nitrogen after in vivo passage in 3-month-old BALB/c mice, according to a previously described protocol [29]. Female BWF1 mice (30 weeks old) were infected by i.p. injection of 10^6^ parasitized erythrocytes obtained from an infected mouse of the same strain, as previously described (28). Parasitemia was monitored by Giemsa-stained thin blood smears. The experimental female BWF1 mice with lupus were assigned to 3 groups (10 mice/group) as follows: group (I), lupus non-infected; group (II), lupus infected with live malaria parasite; and group (III), lupus infected with gamma-irradiated malaria parasite. Group III was infected i.p. with 10^6^ gamma-irradiated red blood cells (iRBCs) infected with *P*. *chabaudi*. Prior to injection, the blood cells were exposed to a dose of 200 Gy gamma-radiation from a Gamma Cell 200 Irradiator (Atomic Energy of Canada, Ltd., Ottawa, Canada) utilizing a ^60^Co source located at the Research Center of the College of Science, King Saud University, Saudi Arabia. This radiation dose was applied based on experiments conducted by Ferreira-da-Cruz et al. [30], which provided evidence that a 200-Gy gamma-irradiation dose abolishes the original replication of erythrocytic forms of the Palo Alto *P*. *falciparum* strain, most likely by inactivating their infectivity. According to their data, 100- or 150-Gy irradiation doses were unable to inactivate the parasite, despite the reduction of parasitemia. Another group of control non-lupus mice (10 mice) was used. All animals were sacrificed at day 14 post-infection. At this time point, live malaria parasite infected group exhibited sever hypokinesia, anorexia and hematuria while other groups didn’t show any of these symptoms.

### Sample collection

Whole blood was collected from the abdominal aorta and immediately transferred into heparinized tubes. The blood was then centrifuged at 4,000 ×*g* for 10 min using a bench-top centrifuge (MSE Minor, England) to remove red blood cells and recover plasma. Plasma samples were separated, collected using dry Pasteur pipettes and stored at −80°C until use. After the plasma isolation, peripheral blood mononuclear cells (PBMCs) were isolated using the Ficoll gradient method. Freshly isolated PBMCs were cultured in RPMI 1640 supplemented with 10% FCS and HEPES (R-10 medium) for at least 4 hours prior to the start of the experiments.

### Determination of plasma cytokine levels

The plasma cytokine profiles were evaluated in triplicate using samples that had been stored at -80°C. The plasma IFN-α level was measured with a commercial ELISA (PBL, Piscataway, NJ) according to the manufacturer’s instructions. Plasma levels of IL-2, IL-4, IL-6, IL-7, IL-12, IL-17, TGF-β, BAFF, IFN-γ, and APRIL were measured by ELISA using the rat Bio-Plex cytokine assay kit (Bio-Rad, Hercules, CA) according to the manufacturer’s instructions.

### Determination of auto-reactive antibodies

Serum Ig levels and anti-dsDNA Abs levels were determined by ELISA according to the manufacturer's instructions. Standard curves for Ig were established using serial dilutions of purified murine IgM, IgG2a, or IgG3 (Sigma-Aldrich, St. Louis, MO), and the data are expressed in micrograms per milliliter.

### Flow cytometry

Cell surface antigen expression on isolated PBMCs was determined by single-parameter fluorescence-activated cell sorter (FACS) analysis with the following monoclonal antibodies (mAbs): (i) FITC-conjugated anti-CD45R/B220, PE-conjugated anti-CCR6, anti-CCR7, anti-CXCR4, anti-CXC5 and PE-conjugated isotype-matched control mAbs (all purchased from R&D Systems, France) and (ii) PE-conjugated anti-CD45R/B220 (BD Biosciences), Alexa Fluor 488 phalloidin (Sigma-Aldrich) and carboxyfluorescein succinimidyl ester (CFSE, Invitrogen). A FACSCalibur flow cytometer (BD-Pharmingen) was used for data acquisition and analysis. After gates were set to include only viable cells, 10^4^ events per sample were collected and analyzed. For each marker, the threshold of positivity was defined relative to the nonspecific binding observed in the presence of the appropriate isotype control mAb.

### F-actin polymerization assay

Isolated PBMCs were cultured for 12 hours in R-10 before the F-actin polymerization test. The cells were first stained with PE-conjugated anti-CD45R/B220 mAb to discriminate between B- and T-cell populations before being subjected to the F-actin polymerization test. Intracellular F-actin polymerization was assessed as previously described [31]. Briefly, the cells were harvested and resuspended (4 x 10^6^/ml) in HEPES-buffered RPMI-1640 at 37°C with or without CXCL12 (500 ng/ml). At the indicated times, the cell suspensions (100 μl) were added to 400 μl of assay buffer containing 4 x 10^7^ FITC-labeled phalloidin, 0.5 mg/ml L-α-lysophosphatidylcholine (both from Sigma-Aldrich) and 4.5% formaldehyde in PBS. The fixed cells were analyzed by flow cytometry, and the mean fluorescence intensity (MFI) was determined for each sample. The percent change in MFI was calculated for each sample at each time point according to the following formula: (1-(MFI before the addition of CXCL12/MFI after the addition of CXCL12)) × 100.

### *In vitro* migration assay

The chemokine-dependent migration of PBMCs isolated from the different groups of mice was measured with an *in vitro* two-chamber migration assay (using Transwell plates purchased from Costar, Cambridge, MA) followed by flow cytometry analysis. Briefly, 600 μl of migration buffer, alone or supplemented with CCL20, CCL21, CXCL12 and CXCL13 (all at 500 ng/ml; R&D Systems), was added to the lower chamber, and 10^4^ cells suspended in migration buffer were added to the upper chamber. The plates were then incubated for 3 hours at 37°C, and the input cells and the transmigrated cells were centrifuged, stained with FITC-conjugated anti-CD45R/B220 mAb, fixed in 300 μl of 1x PBS containing 1% formaldehyde and counted for 60 seconds by flow cytometry with a FACSCalibur flow cytometer (BD-Pharmingen). The migration percentage was calculated as the percentage of input cells that migrated to the lower chamber. To calculate the percentage of specific migration induced by chemokines, the percentage of cells migrating to medium alone was subtracted from the percentage of cells migrating to the medium containing the chemokines.

### CFSE proliferation assay

Isolated PBMCs from the different groups of mice were harvested, washed twice in PBS and stained with 0.63 μM Carboxyfluorescein succinimidyl ester (CFSE) (Molecular Probes, Eugene, OR) for 8 minutes at room temperature. The residual CFSE was removed by three washes with PBS. The CFSE-labeled cells were seeded in 6-well plates, stimulated with IL-4 and CD40L, and grown for 4 days in cell culture medium. After 4 days in culture, the cells were collected, stained with PE-conjugated anti-CD45R/B220 mAb and fixed in 300 μl of 1x PBS containing 1% formaldehyde. The CFSE fluorescence intensity was measured by flow cytometry with a FACSCalibur flow cytometer (BD-Pharmingen).

### Western blot analyses

Western blot analyses were performed as previously described [32–34]. Whole-cell lysates were prepared from the PBMCs isolated from each group in RIPA buffer (20 mM Tris-HCl, pH 7.5, 120 mM NaCl, 1.0% Triton X-100, 0.1% SDS, 1% sodium deoxycholate, 10% glycerol, 1 mM EDTA and 1% protease inhibitor cocktail; Roche). Following centrifugation at 16,000 ×*g* for 15 min at 4°C, the protein concentration of each supernatant was determined using a protein assay kit (Bio-Rad, Hercules, CA). Equal amounts of each whole-cell protein lysate (50 μg) were mixed with reducing sample buffer (0.92 M Tris-HCl, pH 8.8, 1.5% SDS, 4% glycerol and 280 mM 2-mercaptoethanol) and separated by discontinuous SDS-PAGE. The proteins were then transferred onto nitrocellulose membranes using a Bio-Rad Trans-Blot electrophoretic transfer device. Next, the membranes were blocked for 1 hour at room temperature with 1% BSA or 5% skim milk dissolved in TBS (20 mM Tris-HCl, pH 7.4 and 150 mM NaCl) supplemented with 0.1% Tween 20 and then incubated in the same blocking buffer with anti-phospho-AKT, anti-phospho-ERK anti-phospho-P38, anti-phospho-IκB-α, anti-total AKT, anti-total ERK, anti-total P38, IκB-α or anti-β-actin antibodies (1:1,000; Santa Cruz Biotechnology). The blots were thoroughly rinsed and then incubated with an HRP-labeled species-matched secondary antibody for 1 hour. Protein bands were detected by enhanced chemiluminescence (ECL, SuperSignal West Pico Chemiluminescent Substrate, Perbio, Bezons, France), and the ECL signals were recorded on Hyperfilm ECL. To quantify the protein band intensities, the films were scanned, saved as TIFF files and analyzed using NIH ImageJ software.

### Statistical analyses

The data were tested for normality using the Anderson-Darling test and for homogeneity variances prior to further statistical analysis. The data were normally distributed and are expressed as the means ± standard error of the mean (SEM). Significant differences among the groups were analyzed by one- or two-way ANOVA followed by Bonferroni’s test for multiple comparisons using PRISM statistical software (GraphPad Software). The data were reanalyzed by one- or two-way ANOVA followed by Tukey’s post-test, using SPSS software, version 17. Differences were considered statistically significant at P < 0.05. *P < 0.05, lupus vs. control non-lupus; ^#^P < 0.05, lupus + live malaria parasite vs. lupus; ^+^P < 0.05, lupus + dead malaria parasite vs. lupus.

## Results

### Infection with malaria parasite improves and partially restores plasma cytokine levels in lupus mice

We first monitored the plasma cytokine levels of type T_H_1 (IL-2, IL-12, and IFN-γ), type T_H_2 (IL-4 and IL-6), type T_H_17 (IL-17), hematopoietic growth factor (IL-7), transforming growth factor beta (TGF-β), interferon alpha (IFN-α) and B cell activating factors (BAFF and APRIL) in control non-lupus healthy mice and in the 3 experimental groups of lupus mice before and after infection with either live or gamma-irradiated (dead) malaria parasite. The accumulated data from 3 individual mice from each group are shown in Table 1. We observed that lupus mice had aberrant and significantly elevated levels of IL-4, IL-6, IL-7, IL-12, IL-17, IFN-α, IFN-γ, TGF-β, BAFF and APRIL compared with the control non-lupus healthy mice (* P < 0.05). Interestingly, infecting lupus mice with the live parasite partially and significantly decreased the levels of plasma mediators (^#^ P < 0.05; n = 3) compared with the non-infected lupus mice. By contrast, infecting lupus mice with gamma-irradiated malaria parasite did not affect the levels of any of these plasma mediators (n = 3).

**Table 1:** Modulation of plasma cytokine levels in lupus mice after infection with malaria parasite.

	Cytokine levels (pg/ml)	Control	Lupus	Lupus + live malaria	Lupus + irradiated malaria
**T**_**H2**_	**IL-4**	47±3.9	73±6.2*	55±5.1* #	71±7.3
	**IL-6**	24±3.2	77±8.8*	44±5.5* #	72±8.2
**T**_**H1**_	**IL-2**	142±11.7	45±7.2*	93±9.9* #	52±5.1
	**IL-12**	66±7.4	88.6±9.4*	82.8±9.2*	90±10.2
	**IFN-γ**	19±3.1	61.1±7.9*	42.8±5.4* #	57±5.4
**T**_**H17**_	**IL-17**	21±3.7	57.6±6.5*	29.5±3.1* #	59±5.9
	**TGF-β**	14.4±1.8	44.3±7.1*	31.2±3.7* #	42.9±6.4
**Hematopoietic growth factor**	**IL-7**	73±0.2	132±14.4*	95±7.2* #	136±14.1
	**IFN-α**	14±2.4	49±5.9*	25.6±3.9* #	45±6
**B cell activating factors**	**BAFF**	13.6±3.2	68.8±9.2*	38.4±6.4* #	66±7.2
	**APRIL**	9.3±2.4	35.6±5.2*	19.6±3.66* #	32.8±4.8

The levels of plasma T_H_1 (IL-2, IL-12 and IFN-γ), T_H_2 (IL-4 and IL-6), T_H_17 (IL-17), hematopoietic growth factor (IL-7), transforming growth factor beta (TGF-β), interferon alpha (IFN-α) and B cell activating factors (BAFF and APRIL) cytokines were measured in non-lupus control mice and in the 3 groups of lupus mice before and after infection with either live or gamma-irradiated malaria parasite. The experiment was performed in triplicate, and the results are presented as the cytokine levels (pg) per ml of plasma and are expressed as the means ± SEM (n = 3).

*P < 0.05, lupus vs. non-lupus control;

^#^P < 0.05, lupus + live malaria parasite vs. lupus (ANOVA with Tukey’s post-test).

### Infecting lupus mice with live malaria parasite significantly restores the levels of IgG2a and IgG3 with no effect on IgM

**Fig 1:**
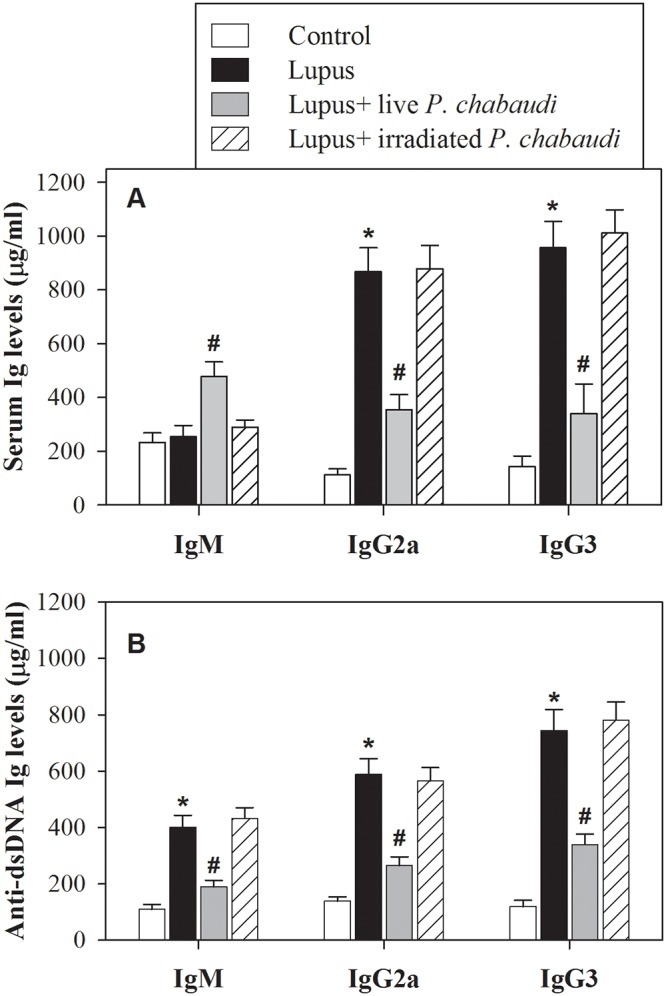
Effects of malaria parasite infection on serum Ig levels. The levels of serum Ig (**A**) and anti-dsDNA Ig (**B**) IgM, IgG2a and IgG3 were quantitated by ELISA in non-lupus control mice (open bars), lupus mice (closed black bars), lupus mice infected with live malaria parasite (closed gray bars) and lupus mice treated with gamma-irradiated malaria parasite (hatched bars). The combined data from 5 mice from each group (n = 5) are shown, and the results are expressed as the mean level of Ig (μg) per ml of serum ± SEM. *P < 0.05, lupus vs. control non lupus; ^#^P < 0.05, lupus + live malaria vs. lupus; ^+^P < 0.05, lupus + dead malaria vs. lupus.

Using ELISA, we tested the levels of serum and anti-dsDNA IgM, IgG2a and IgG3 in the four groups of mice because the generation of these antibodies in patients with lupus causes glomerulonephritis. The accumulated results from 5 mice from each group (n = 5) are shown in Fig 1. The serum levels of IgG2a and IgG3 significantly increased in lupus mice, while no differences in IgM levels were observed between the lupus mice and the non-lupus control group (* P < 0.05) Fig 1A. Infection of these lupus mice with live malaria parasite significantly decreased the levels of IgG2a and IgG3 autoantibodies compared with the non-infected lupus group (^#^ P < 0.05, n = 5). Additionally, lupus mice infected with gamma-irradiated malaria parasite exhibited no significant effect on the levels of serum IgM, IgG2a and IgG3. In contrast, serum levels of IgM were significantly higher in the lupus mice infected with live malaria parasite than in the non-infected lupus group (^#^ P < 0.05, n = 5). Most importantly, lupus mice exhibited a significant increase in the levels of anti-dsDNA Abs compared with non-lupus control mice (* P < 0.05, n = 5) Fig 1B. Interestingly, infection of lupus mice with live malaria parasite, but not with gamma-irradiated malaria parasite, significantly decreased the levels of all anti-dsDNA Abs compared with the non-infected lupus group (^#^ P < 0.05, n = 5).

### Lupus mice infected with live malaria parasite exhibited a restored surface expression of CXCR4 on B cells

**Fig 2:**
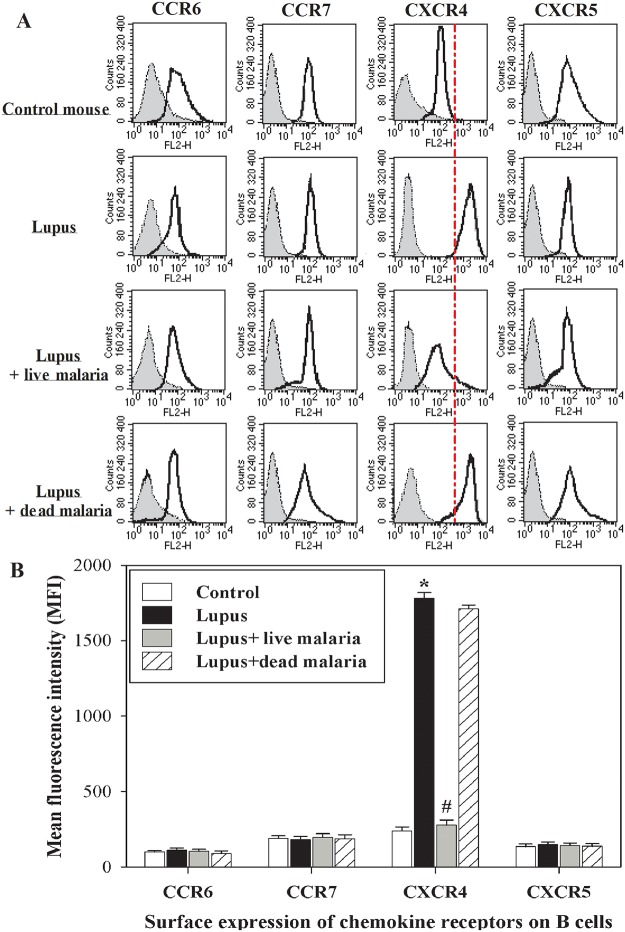
Surface expression of chemokine receptors in B cells. The surface expression levels of CCR6, CCR7, CXCR4 and CXCR5 were analyzed by flow cytometry in B cells. PBMCs were isolated from non-lupus control mice, lupus mice, lupus mice infected with live malaria parasite and lupus mice treated with gamma-irradiated malaria parasite. Cells were harvested, cultured overnight at 37°C in culture medium and then washed twice in PBS. Cells were first stained for 30 minutes at 4°C with the directly conjugated monoclonal antibody (mAb) CD45R/B220-fluorescein isothiocyanate (FITC). Then, they were stained for 30 minutes at 4°C with the following directly conjugated mAbs: CCR6-phycoerythrin (PE), CCR7-PE, CXCR4-PE and CXCR5-PE (depicted with open, bold-line histograms) as well as an isotype-matched control (IgG) (closed, thin-line histograms). Cells were then washed in PBS and fixed in fixation buffer, and the analyzed cell populations were gated on the viable CD45R/B220-FITC positive population in the lymphocytes area. One representative experiment is shown (**A**).The accumulated results (n = 5) for the MFI of each of the chemokine receptors are expressed as the MFI ± SEM in non-lupus control mice (open bars), lupus mice (closed black bars), lupus mice infected with live malaria parasite (closed gray bars) and lupus mice treated with gamma-irradiated malaria parasite (hatched bars). *P < 0.05, lupus vs. control non lupus; ^#^P < 0.05, lupus + live malaria vs. lupus; ^+^P < 0.05, lupus + dead malaria vs. lupus.

Chemokines and chemokine receptors are used as therapeutic targets in lupus nephritis; therefore, we assessed the surface expression of the most important chemokine receptors (CCR6, CCR7, CXCR4 and CXCR5) on B lymphocytes from all four groups by flow cytometry. In a representative experiment, we found that, unlike CCR6, CCR7 and CXCR5, the surface expression of CXCR4 was substantially up-regulated on the surface of B cells of lupus mice compared with the control non-lupus mice **(** Fig 2A **)**. Infecting lupus mice with live, but not dead, malaria parasite restored the surface expression of CXCR4 on the B cells. The accumulated results (n = 5) for the MFI of each chemokine receptor are expressed as the MFI ± SEM (Fig 2B). In this context, we observed that the surface expression of CXCR4 on the B cells of lupus mice was significantly up-regulated compared with the non-lupus control group (* P < 0.05). Surprisingly, the surface expression of CXCR4 on the B cells of lupus mice infected with live malaria parasite, but not gamma-irradiated malaria parasite, was significantly restored compared with the non-infected lupus group (^#^ P < 0.05, n = 5). In contrast, infecting lupus mice with live or gamma-irradiated malaria parasite had no effects on the surface expression of CCR6, CCR7 or CXCR5.

### Infecting lupus mice with live malaria improved chemokine-mediated actin polymerization and chemotaxis in B cells

**Fig 3:**
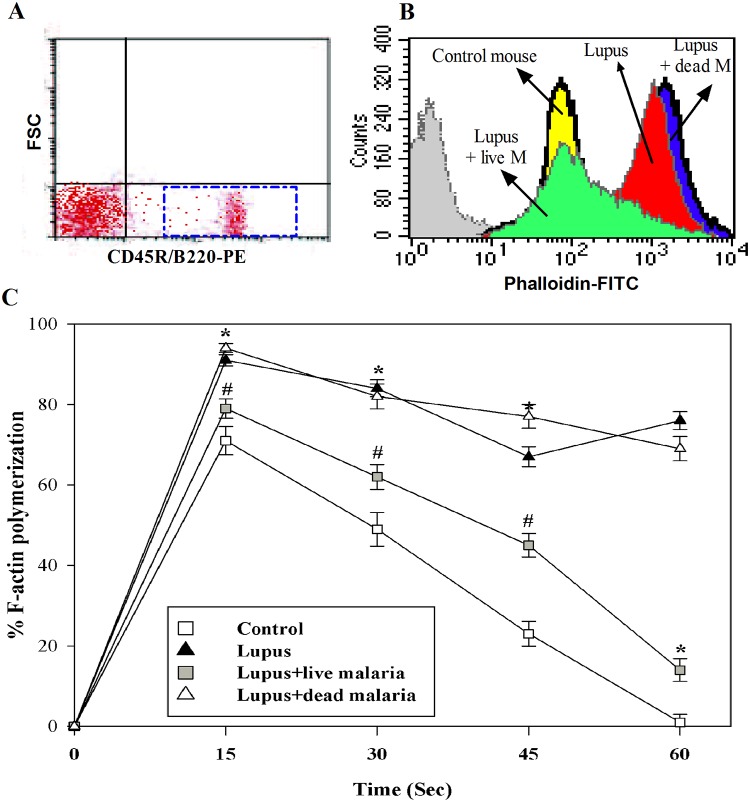
Modulation of chemokine-mediated actin polymerization in B cells of malaria-infected lupus mice. PBMCs were subjected to an F-actin polymerization assay after CXCL12 stimulation at the indicated time intervals (every 15 seconds), and the results were quantified by flow cytometry. (**A**) The analyzed cell populations were gated to the viable CD45R/B220-PE-positive population in the lymphocyte area according to forward and side scatters (FSC/SSC). (**B**) One representative experiment showing the different histograms at 30 seconds of stimulation with CXCL12. (**C**) The data from the different experiments (n = 5) are expressed as the percentage change in MFI ± SEM. *P < 0.05, lupus vs. control non lupus; ^#^P < 0.05, lupus + live malaria vs. lupus; ^+^P < 0.05, lupus + dead malaria vs. lupus.

Chemokine-mediated actin polymerization was monitored in B cells in the three experimental lupus groups and in the non-lupus control mice. PBMCs were stimulated with CXCL12 every 15 seconds, and actin polymerization was monitored using phalloidin-FITC and flow cytometry analysis. The analyzed cell populations were gated to the viable CD45R/B220-PE-positive population (B cells) in the lymphocyte area, according to forward and side scatters (FSC/SSC) (Fig 3A). CXCL12-mediated actin polymerization at 30 seconds is illustrated in histograms from one representative experiment (Fig 3B). CXCL12 aberrantly mediated actin polymerization in the lupus mouse (red histogram) compared with the non-lupus control mouse (yellow histogram). Additionally, the lupus mouse infected with live (green histogram), but not gamma-irradiated malaria (blue histogram) parasite, exhibited a marked restoration in CXCL12-mediated actin polymerization. Accumulated data from the different experiments (n = 5) are expressed as the percentage change in MFI ± SEM. We found that the percentage of CXCL12-mediated actin polymerization every 15 seconds was significantly and aberrantly increased in lupus mice compared with the non-lupus control group (* P < 0.05) (Fig 3C). Interestingly, lupus mice infected with live malaria parasite exhibited a significant reduction in CXCL12-mediated actin polymerization compared with the lupus group (^#^ P < 0.05, n = 5). In contrast, when compared throughout the stimulation time, treating lupus mice with gamma-irradiated malaria parasite did not affect CXCL12-mediated actin polymerization compared with the lupus group.

**Fig 4:**
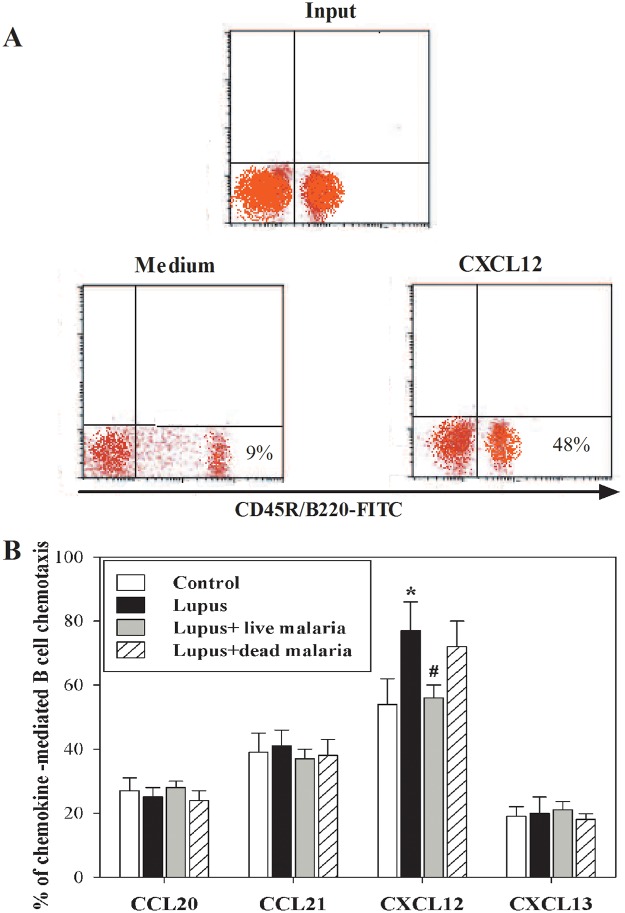
Modulation of chemokine-mediated chemotaxis of B cells of malaria-infected lupus mice. PBMCs were subjected to migration assays in response to CCL20, CCL21, CXCL12, and CXCL13. (**A**) Input and migrated cells were stained with CD45R/B220-FITC. The cells were then counted for 60 seconds by flow cytometry to calculate the percentage of cells that migrated nonspecifically (based on the number of cells that migrated in medium alone) or specifically (based on the number of cells that migrated in medium plus CXCL12). To calculate the percentage of specific migration induced by chemokines, the percentage of cells migrating to medium alone was subtracted from the percentage of cells migrating to the medium containing the chemokines. (**B**) The data from the different experiments are expressed as the mean percentage of chemokine-mediated specific migration of B cells ± SEM in non-lupus control mice (open bars), lupus mice (closed black bars), lupus mice infected with live malaria parasite (closed gray bars) and lupus mice treated with gamma-irradiated malaria parasite (hatched bars). *P < 0.05, lupus vs. control non lupus; ^#^P < 0.05, lupus + live malaria vs. lupus; ^+^P < 0.05, lupus + dead malaria vs. lupus.

Because actin polymerization in response to chemokines is an initial step in cell locomotion, we assessed the chemotactic response of B cells toward CCL20, CCL21, CXCL12, and CXCL13 using a chemotaxis assay and flow cytometry analysis. Input and PBMCs that migrated to medium alone or medium supplemented with chemokines were stained with CD45R/B220-FITC (Fig 4A). In one representative experiment, the percentage of migrated CD45R/B220-FITC-positive B cells from a non-lupus control mouse was 9% in medium alone versus 48% in medium plus CXCL12. Therefore, the specific migration of B cells toward CXCL12 was 39% (48%- 9%). The data from the different experiments (n = 5 mice from each group) are expressed as the mean percentage of chemokine-mediated specific migration of B cells ± SEM in the three experimental lupus groups as well as in the non-lupus control mice. These data reveal that the percentage of specifically migrated B cells was significantly (*P < 0.05) increased in lupus mice compared with the non-lupus control group (Fig 4B). However, unlike the lupus mice treated with dead malaria parasite, the lupus mice infected with live malaria parasite exhibited a significant restoration (^#^P < 0.05) in the percentage of B cell chemotaxis compared with the non-infected lupus mice.

### Infecting lupus mice with live malaria parasite improved the proliferative capacity of B cells

Aberrant proliferation of autoimmune B cells is of potential importance in the pathogenesis of SLE. Therefore, we monitored the proliferative capacity of B cells in response to IL-4 and CD40L in the three experimental lupus groups as well as in the non-lupus control mice using CFSE assays and flow cytometry analysis.

**Fig 5:**
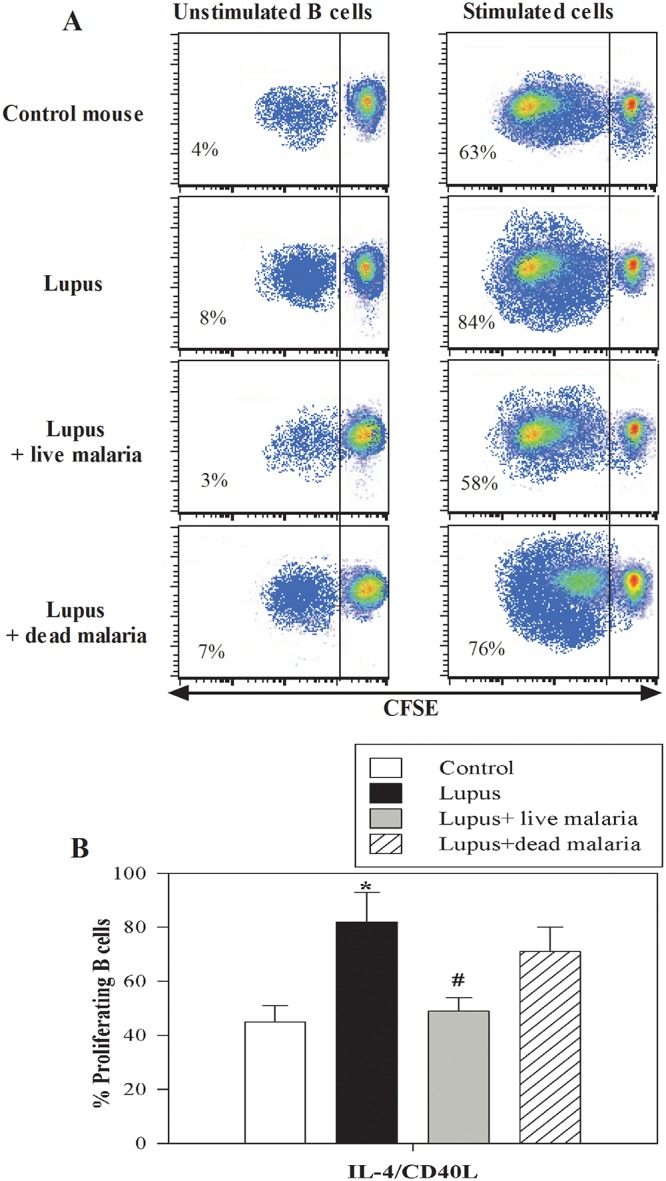
Altered proliferative capacity of B cells in lupus mice after infection with malaria parasite. The proliferative capacity of B cells in response to IL-4 and CD40L stimulation was evaluated using CFSE assays and flow cytometry. (**A**) One representative experiment showing the analysis of CFSE-stained B cells (after gating to viable cells); the percentage of proliferating cells (CFSE-lo) is indicated for each panel before and after infection of lupus mice with live or gamma-irradiated malaria parasite. (**B**) The data from the different experiments (n = 5) are expressed as the mean percentage of proliferating cells ± SEM in response to IL-4 and CD40L stimulation in non-lupus control mice (open bars), lupus mice (closed black bars), lupus mice infected with live malaria parasite (closed gray bars) and lupus mice treated with gamma-irradiated malaria parasite (hatched bars). *P < 0.05, lupus vs. control non lupus; ^#^P < 0.05, lupus + live malaria vs. lupus; ^+^P < 0.05, lupus + dead malaria vs. lupus.

The CFSE-labeled cells were either stimulated with IL-4 and CD40L or maintained unstimulated and then were grown for 4 days in cell culture medium. After 4 days in culture, the cells were stained with PE-CD45R/B220 mAb and analyzed for their proliferative capacity using flow cytometry. One representative experiment is shown for the analysis strategy of CFSE-stained B cells (after gating to viable cells), and the percentage of proliferating cells (CFSE-lo) is indicated for each panel in the different treatment groups. We found that the percentage of proliferating B cells following IL-4 and CD40L stimulation increased from 4% to 63% (59% specific proliferation) in the non-lupus control mice, from 8% to 84% (76% specific proliferation) in lupus mice, from 3% to 58% (55% specific proliferation) in lupus mice infected with live malaria parasite and from 7% to 76% (69% specific proliferation) in lupus mice treated with dead malaria parasite (Fig 5A). Likewise, we noticed that lupus mouse exhibited marked elevation in the proliferative capacity of B cells compared with non-lupus control mice. Additionally, lupus mice infected with live, but not dead, malaria parasite exhibited a clear restoration in B cell proliferation. The data from the different experiments (n = 5) are expressed as the mean percentage of proliferating cells ± SEM in response to IL-4 and CD40L stimulation in all of the experimental groups. In this context, our data reveal that the proliferative capacity of the B cells was significantly elevated in lupus mice compared with the control group (*P < 0.05) (Fig 5B). Interestingly, when lupus mice were infected with live but not dead malaria parasite, they exhibited a significant restoration of B cell proliferative capacity compared with the lupus group (^#^P < 0.05).

### Infecting lupus mice with live malaria parasite induced modulation of the AKT, NFκBα and ERK signaling pathways but not p38

**Fig 6:**
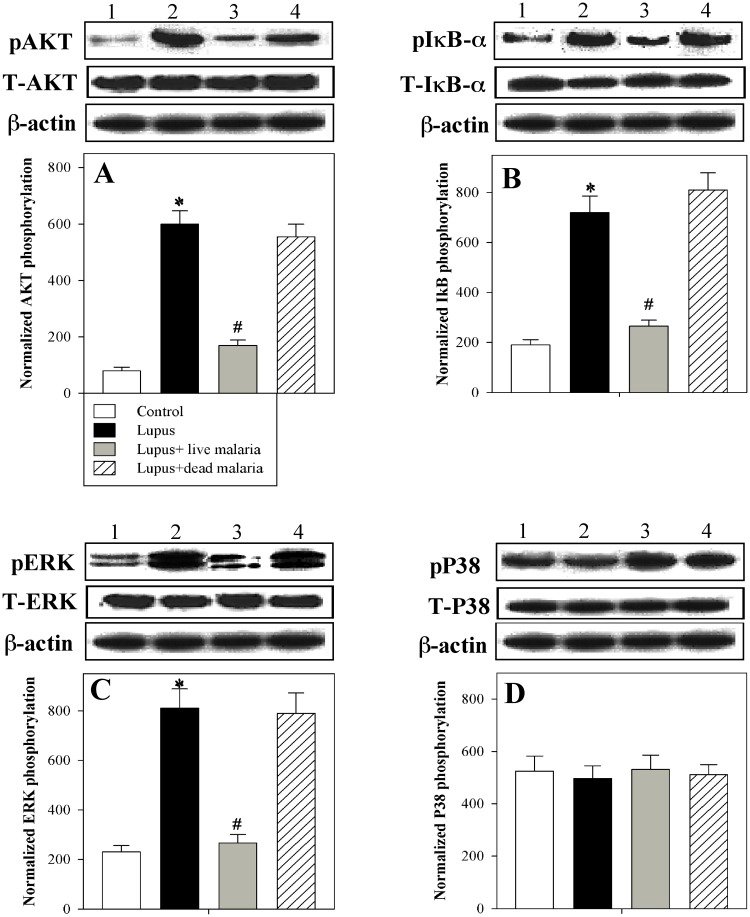
Altered phosphorylation of AKT, ERK, IκB-α and P38 in PBMCs of malaria-infected lupus mice. PBMCs isolated from control non-lupus mice, lupus mice, lupus mice infected with live malaria parasite and lupus mice treated with gamma-irradiated malaria parasite were stimulated with medium or CXCL12. Cells were then lysed and subjected to Western blotting using antibodies recognizing pAKT, pIκBα, pERK and pP38. The protein bands from one representative experiment are shown (**A**). The expression of all indicated proteins was normalized to the total relevant proteins as well as to the total β-actin protein levels. The accumulated results from five animals per group are expressed as the means ± SEM of the normalized values of all proteins in non-lupus control mice (open bars), lupus mice (closed black bars), lupus mice infected with live malaria parasite (closed gray bars) and lupus mice treated with gamma-irradiated malaria parasite (hatched bars). *P < 0.05, lupus vs. control non lupus; ^#^P < 0.05, lupus + live malaria vs. lupus; ^+^P < 0.05, lupus + dead malaria vs. lupus.

The phosphorylation of cellular effectors that play a central role in B cell activation and autoreactivity, such as AKT, IκBα, ERK and p38, was investigated in PBMCs using Western blot analysis. The immunoblots of one representative experiment are shown in Fig 6. The expression of all indicated proteins was normalized to the total relevant proteins as well as to total β-actin protein levels. The accumulated results from five animals of each group are expressed as the means ± SEM of the normalized values of all proteins in the four animal groups. We found that the phosphorylation levels of AKT, IκBα and ERK were significantly (*P < 0.05) increased in the lupus mice compared with the non-lupus control group. Interestingly, when lupus mice were infected with live but not dead malaria parasite, they exhibited a significant restoration in the phosphorylation level of AKT, IκBα and ERK compared with the lupus group (^#^P < 0.05). In contrast, no significant differences were observed in the phosphorylation of p38 among the four experimental groups.

## Discussion

Cytokines are essential mediators of intercellular communication and orchestrate the interactions of immune cells during immune responses. In SLE, several cytokines are involved in general immune dysregulation and abnormalities in the signaling pathways of B lymphocytes, which contribute to the development of SLE pathogenesis and autoimmune disease [35]. Thus, cytokine imbalance plays a significant role in the acceleration of lupus-like autoimmune disease. The shifting of Th1 to Th2 immune responses results in B cell hyperactivity and the production of pathogenic auto-antibodies and inflammation [36]. We previously reported that infection of female BWF1 lupus mice with malaria parasite alters the redox state in the kidney and liver tissues and confers protection against lupus nephritis [6]. This knowledge about the cytokine profiles in SLE not only provided new insight into the pathogenesis of SLE but also shed light on various clinical applications. In the current study, we present interesting findings that clarify the molecular mechanisms of SLE severity based on the immune response in the B cells of female BWF1 lupus mice after infection with either live or gamma-irradiated malaria parasite. Our findings revealed increased levels of IL-4, IL-6, IL-7, IL-12, IL-17, IFN-α, IFN-γ, TGF-β, BAFF and APRIL in lupus mice. Indeed, in SLE, cytokines contribute to the development and/or progression of the disease both in mice and in humans [37]. Similar observations have revealed elevated levels of proinflammatory cytokines (IL-17, IL-12), Th2 (IL-4), BAFF and APRIL in patients with SLE [38, 39]. Moreover, soluble, biologically active BAFF is elevated in the serum of a fraction of patients with active SLE [40, 41]. Surprisingly, infecting lupus mice with live malaria parasite, but not gamma-irradiated malaria parasite, partially and significantly decreased their levels of plasma cytokines. Similarly, malarial infection alters the expression of BAFF, thus attenuating memory B cell differentiation into antibody-secreting cells [42]. Therefore, altering plasma cytokine levels by malarial infection represents a potential therapeutic strategy for the treatment of SLE. Our study revealed that infecting lupus mice with live malaria parasite significantly restored the levels of auto reactive antibodies IgG2a and IgG3 with no effect on IgM Abs. Previous studies have shown that lupus is accompanied by B cell activation, elevated serum levels of IgG2a, IgG3 and ant-dsDNA autoantibodies and increased production of cytokines [43, 44]. Our results provide evidences for the restoration of the IgG2a and IgG3 levels as well as decreased anti-dsDNA levels induced by malarial infection may be cross-linked with plasma levels of cytokines. Chemokines and their receptors are essential in the recruitment and positioning of lymphocytes [18]. The analysis of peripheral blood B cells by flow cytometry showed that the surface expression of CXCR4 was up-regulated on the surface of B cells of lupus mice, whereas there was no detectable change in the surface expression of CCR6, CCR7 and CXCR5. Similarly, Wang et al. [45] reported that CXCR4/CXCL12 over-expression plays a pivotal role in the pathogenesis of lupus. This increase in CXCR4 expression may be due to the occurrence of inflammation in lupus mice. In contrast, another study reported that CXCR4 expression on circulating B cells was significantly lower in SLE patients than in healthy controls and that this decrease in CXCR4 expression could be due to changes in the proportion of B cell subsets or variations in expression levels within a subset [22]. Strikingly, our study demonstrated that lupus mice infected with live malaria parasite exhibit a restored surface expression of CXCR4 on B cells. These findings suggest that chemokine receptors, particularly CXCR4, are promising therapeutic targets in the fight against SLE. In this context, a recent study demonstrated that changes in antigen stimulation from a successive, strong immune stimulus like *P*. *falciparum* infection may lead to changes in the overall B cell population [46]. Furthermore, prior studies have demonstrated alterations in the proportions of B cell subsets in the peripheral circulation following *Plasmodium* infections in children [47]. Additionally, blocking the CXCR4/CXCL12 interaction causes an increase in circulating parasitemia, suggesting a pivotal role for CXCR4/CXCL12 signaling during malarial infection [48]. Moreover, a recent study reported that the CXCR4/CXCL12 axis controls auto-immunity in lupus patients following influenza vaccine [49]. Because the B cells of lupus mice exhibited high expression levels of CXCR4, we monitored the B cell responsiveness to CXCL12 in term of actin polymerization, chemotaxis, proliferation and signaling. We observed that lupus mice infected with live malaria parasite, but not gamma-irradiated malaria parasite, exhibited a significant reduction in CXCL12-mediated actin polymerization compared with the non-lupus control group. Actin depolymerization induces altered lipid raft clustering and ERK activation [50], suggesting that SLE can be inhibited by involving the actin cytoskeleton. Therefore, chemokines and their receptors affect the inflammatory development and progression of B-cell-mediated autoimmune diseases, including SLE [51, 52]. Our data also revealed that, unlike treating lupus mice with dead malaria parasite, lupus mice infected with live malaria parasite exhibited a significant restoration in B cell chemotaxis. This modulation in B cell chemotaxis may be due to altered CXCR4 expression induced by malarial infection [53]. Consequently, there may be a correlation among CXCR4 expression, B cell chemotaxis and SLE activity. Notably, the B cell proliferative capacity was significantly elevated in lupus mice. Interestingly, when lupus mice were infected with live but not dead malaria parasite, they exhibited a significant restoration of B cell proliferation. A previous study identified BAFF as one of the critical factors controlling B cell maturation, tolerance, and malignancy [54], suggesting that B cell proliferation is regulated by BAFF. Additionally, IL-7 plays several important roles during B cell development, including promoting the proliferation and survival of B cell progenitors and maturation during the pro-B to pre-B cell transition [55]. These roles suggest a link among plasma cytokine levels, B cell proliferation and SLE severity with or without malarial infection. We previously reported that chemokines such as CCL20, CCL21 and CXCL12 induce actin polymerization and chemotaxis in B lymphocytes through the activation of PI3K/AKT, NF-κB, PLC, ERK and P38MAPK signaling [56]. In this study, our data revealed that infecting lupus mice with live malaria parasite regulated the AKT, NFκBα and ERK signaling pathways but not p38. Similarly, previous studies have reported that, during SLE disease progression, the signaling cascades involving PI3K/AKT, MAPKs (ERK, JNK, p38) and regulators of the nuclear translocation of NF-κB (IκBs) are critically involved in B cell differentiation and the production of autoantibodies [57, 58]. Taken together, our data reveal that infecting lupus mice with malaria parasite confers protection against lupus through the direct attenuation of B cell autoreactivity, providing a new therapeutic strategy to control SLE.
